# Adaptive and innate immune responses in multiple sclerosis with anti-CD20 therapy: Gene expression and protein profiles

**DOI:** 10.3389/fneur.2023.1158487

**Published:** 2023-04-24

**Authors:** Chloe C. Fong, Julian Spencer, Quentin Howlett-Prieto, Xuan Feng, Anthony T. Reder

**Affiliations:** Department of Neurology, University of Chicago Medicine, Chicago, IL, United States

**Keywords:** anti-CD20 therapy, B cell, multiple sclerosis, ocrelizumab, T cell, TLR

## Abstract

**Background:**

Anti-CD20 is a highly effective therapy for multiple sclerosis (MS), a disease with multiple abnormalities in function of B and T cells and innate immune cells. Anti-CD20 therapy depletes B cells, which alters antibody production and has diverse effects on B cell immunity. These changes potentially affect immunity beyond B cells in MS.

**Objective:**

Determine if anti-CD20 therapy effects non-B cell, as well as B cell, gene expression, and serum protein levels.

**Methods:**

Samples were collected from 10 healthy controls and from clinically stable relapsing–remitting MS – 10 untreated, 9 interferon-*β*-treated, and 15 ocrelizumab-treated patients were studied before, and 2  weeks and 6  months after, the first anti-CD20 infusion. Peripheral blood mononuclear cells (PBMC) were analyzed with sensitive, 135,000-transcript RNA expression microarrays, using stringent criteria. Gene expression was compared to 43 MS-relevant serum immune and neurotrophic proteins, using multiplex protein assays.

**Results:**

Anti-CD20 therapy reduced expression of 413 total genes and 185 B-cell-regulated genes at 2  weeks vs. pre-therapy. Expression of 19 (15%) of these B cell genes returned toward baseline by 6  months, including genes for the B cell activation protein, CD79A, and for immunoglobulin A, D, and G heavy chains. Expression pathways for Th17 and CD4 regulatory T-cell (Treg) development, differentiation, and proliferation also quieted. In contrast, expression increased in Th1 and myeloid cell antiviral, pro-inflammatory, and toll-like receptor (TLR) gene pathways.

**Conclusion:**

These findings have clinical implications. B cell gene expression diminishes 2  weeks after anti-CD20 antibody infusion, but begins to rebound by 6  months. This suggests that the optimum time for vaccination is soon before reinfusion of anti-CD20 therapy. In addition, at 6  months, there is enhanced Th1 cell gene expression and induction of innate immune response genes and TLR expression, which can enhance anti-viral and anti-tumor immunity. This may compensate for diminished B cell gene expression after therapy. These data suggest that anti-CD20 therapy has dynamic effect on B cells and causes a compensatory rise in Th1 and myeloid immunity.

## Introduction

Anti-CD20 monoclonal antibodies potently reduce MRI lesions and relapses, and slow accumulation of disability in relapsing–remitting multiple sclerosis (RRMS) and primary progressive MS (PPMS) (1, 2). Anti-CD20 therapy depletes pre-B cells, naïve B cells, and mature B cells, but spares CD20-negative immature B cell precursors and mature plasma cells.

A pathogenic role for B cells in MS is suggested by presence of activated pro-inflammatory B cells (3), elevated virus-specific antibodies in blood and CSF (4, 5), and excessive mitogen-activated antibody production (6). The mechanism of action of anti-CD20 therapy in MS was originally presumed to be through interruption of antibody-mediated immunity (7). Anti-CD20 therapy also modifies other B cell and non-B cell functions, including (1) interruption of antigen presentation by B cells (8), abnormally enhanced by their high CD80 costimulatory molecule expression (9); (2) reduction of inflammatory B cell cytokines, IL-6, lymphotoxin, and GM-CSF (8); (3) depletion of germinal center-like areas in meninges (10); (4) depletion of activated EBV-infected B cells (4); (5) depletion of CD20^dim^ CD4 and CD8 cells; and (6) reversal of T-cell dysregulation and brain damage from inflammatory T cells and macrophages.

Clinical interventions demonstrate that the B cell role in MS is complex. Although targeted at B cell immunity, anti-BAFF/BLyS and anti-APRIL therapies induce exacerbations (11). In contrast, anti-CD20 MS therapies prevent inflammation. They raise regulatory CD8 + T cells from subnormal to supranormal levels over 18 months (12), deplete a small population of cytolytic CD20^dim^, CD8+ T cells that recognize myelin basic protein (13) and deplete CD20^dim^ CD4 and CD8 cells that secrete pro-inflammatory cytokines and predict MS disease activity (14, 15), and reduce pro-inflammatory IL-12 and increase anti-inflammatory IL-10 production by monocytes (8). However, comprehensive effects of anti-CD20 therapy on gene expression and immune proteins in non-B cells are not defined.

We hypothesized that anti-CD20 therapy effects T cells and innate immunity, in addition to depletion of B cells. We profiled B cell and non-B-cell gene expression and protein signatures from MS peripheral blood mononuclear cells (PBMC) and serum before and after ocrelizumab treatment compared to therapy-naïve and IFN-*β*-treated MS patients.

## Methods

### Subjects and clinical data

Subjects included 34 clinically stable MS: 15 ocrelizumab-treated, 9 interferon (IFN)-*β*-treated, and 10 therapy-naïve (No Rx). Patients were age-matched with 10 healthy controls (HC) (Table 1). Stable MS was defined as no clinical attacks for >2 months or progression of >0.5 EDSS point in the past 6 months. The time since last exacerbation in the ocrelizumab group averaged 33.5 ± 13.3 months, based on detailed history, exams, and available MRI. Exclusion criteria included exacerbation within prior 2 months, infection, stroke history, and clinically significant cardiovascular disease. During the study, there were no clinical exacerbations. MRI was not performed in ocrelizumab-treated patients because new lesions, typically solitary, appear in <1% of MRI scans and do not lead to changes in therapy (1, 2).

**Table 1:** Subject demographics and baseline characteristics.

	HC (*n* = 10)	No Rx MS (*n* = 10)	IFN-β Rx MS (*n* = 9)	Ocre Rx MS (*n* = 15)
MS type (n)	NA	RRMS (7) SPMS (3)	RRMS (8) SPMS (1)	RRMS (9) SPMS (6)
Age, years (Mean ± SEM)	47.6 ± 5.3	50.1 ± 3.4	50.7 ± 3.3	51.8 ± 3.0
Sex	3 M/7F	1 M/9F	2 M/7F	7 M/8F
Race	6 W/2B/1H/1A	7 W/2B/1H	7 W/2B	12 W/2H/1P
MS Duration, years. (Mean ± SEM)	NA	16.4 ± 4.6	14.7 ± 2.7	20.3 ± 3.4
Months since prior therapy (Washout)	NA	NA	0	2.00 ± 0.86
EDSS (Mean ± SEM)	NA	3.9 ± 1.4	3.2 ± 0.6	3.6 ± 0.6
Vitamin D, ng/ml (Mean ± SEM)	NA	44.6 ± 7.1	40.4 ± 5.2	52.5 ± 5.5
Current Smoking	3	0	1	0
BMI (Mean ± SEM)	26.7 ± 1.9	25.5 ± 1.5	31.2 ± 1.8	29.6 ± 2.3

A, East Asian; B, Black/African ancestry; BMI, Body mass index; EDSS, Extended disability scale score of Kurtzke; F, female; H, hispanic; IFN, interferon-β; n, number; NA, not applicable/available. Ocre, ocrelizumab; P, Filipino; RRMS, relapsing–remitting MS; SEM, standard error of the mean; SPMS, secondary progressive MS; W, white. Values are given as mean ± standard error (SEM). Vitamin D levels were available in 8 No-Rx MS and 8 IFN-MS; normal = 30–100 ng/ml serum. Ocrelizumab-treated patients were studied pre-treatment and 2 weeks and 6 months afterward, before the next infusion. There were no significant differences between groups (See Methods, Subjects, and Clinical data).

Thus, correlations with disease activity would require a sample size of >800.

Ocrelizumab-treated patients in this “real world” study were therapy-naïve (*n* = 2) or had recently stopped other therapies, including IFN-β (*n* = 5), natalizumab (*n* = 4), glatiramer acetate (GA, *n* = 1), teriflunomide (1), and fingolimod (2); therapy washout averaged 2.00 ± 0.86 months. Reasons for change in therapy were injection fatigue (1 IFN-β and 1 GA), positive JC virus titers (3 natalizumab), infusion reaction (1 natalizumab), and perception of worse MS (7 others). There were no rebound exacerbations after discontinuation. Demographic variables were not statistically different, including the sex ratio in ocrelizumab-treated vs. untreated MS (Χ^2^ = 2.2, *p* = 0.15). In the current study, we found no informative differences in protein or gene expression (including Y-RNA) vs. sex ratio, vitamin D level, EDSS, disease duration, smoking, BMI, or between stable SPMS and RRMS patients. The latter two groups were combined for analysis.

Time 0 blood was drawn at 8 AM, followed by the first infusion of 300 mg IV ocrelizumab. Patients were sampled again at 2 weeks, reinfused with 300 mg ocrelizumab, and then sampled at 6 months, before that infusion. Seventy three gene samples and 34 protein samples were analyzed. All subjects signed University of Chicago IRB-approved informed consents.

### Multiplex analysis of serum proteins

Serum was frozen within 1 h of phlebotomy. Multiplex immunoassays of serum proteins included 43 cytokines, chemokines, and neurotrophic proteins curated as relevant to MS (16, 17) and detectable in serum, based on preliminary assays of 280 analytes. Thirty three targets were in a commercially-available 66-plex array, and 10 in a custom array (ProcartaPlex/Thermo Fisher Luminex). Assays were run with duplicate samples according to the manufacturer’s guidelines, and patient subtypes and longitudinal samples were randomized between plates (18).

### PBMC isolation, RNA preparation, and microarray assays

PBMCs were purified with density gradients (Lympholyte, Cedarlane) within 4 h of phlebotomy. Cell lysates were stored at −80°C in “buffer RLT plus” (Qiagen) for <12 months (18, 19).

Total RNA was extracted from frozen PBMC with standard isolation protocols (Qiagen). All samples passed RNA quality Hybridization Controls. Samples were hybridized to next-generation Clariom D microarrays (6.7 million probes of 135,000 transcripts; Thermo Fisher Scientific). Microarray expression has been validated with QuantiGene assays (Thermo Fisher) (19).

### Visualization of differentially expressed genes and gene expression analysis

**Figure 1:**
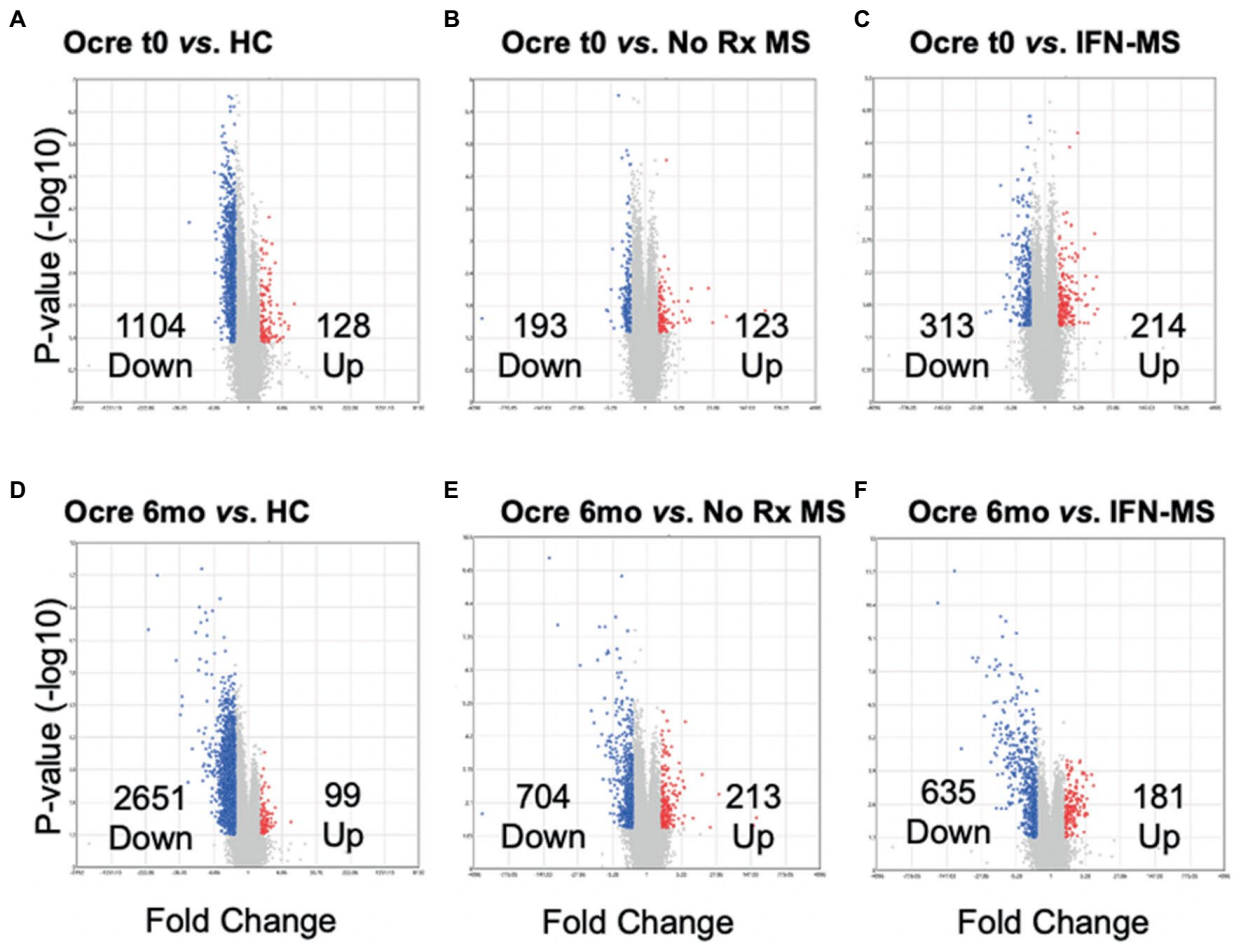
Differentially expressed genes (DEG) in PBMC from 15 MS patients at time 0 and 6  months after ocrelizumab infusion, compared to HC, therapy-naïve (No Rx) MS, and IFN-*β*-treated MS. **(A)** 15 ocrelizumab-treated MS at time 0 have more genes with decreased expression than 10 HC, **(B)** 10 therapy-naïve MS, and **(C)** 9 IFN-*β*-treated MS. Ocrelizumab-treated MS at 6  months have decreased gene expression compared to **(D)** HC, **(E)** therapy-naïve MS, and **(F)** IFN-*β*-treated MS. Red indicates genes upregulated; blue indicates genes decreased in ocrelizumab-treated compared to other groups. Uncharacterized, AceView-identified, genes (gray) excluded from analysis even if fold-change is >|2|.

Raw CEL files were imported, log2-transformed, and normalized. Transcriptome Analysis Console (TAC) 4.02 was used to identify differentially expressed genes (DEGs) over time and between groups (19) When using only TAC filter options, uncharacterized (“AceView-identified”) genes were excluded from further analysis to decrease noise (e.g., Figure 1). Gene lists were also filtered using both TAC and Human Protein Atlas.

Ingenuity Pathways Analysis (IPA) Knowledge Base identified Disease and Function pathways altered during treatment. IPA Downstream Effects Analysis predicted increases or decreases in biological activities and putative effects on diseases and cell biological processes.

### Gene lists for immune cell subsets

*B cell* subset specificity was deconvoluted using a two-step screening process to generate three PBMC expression categories. (1) “B-cell-related” genes were first identified using TAC filters, “*contain Ig,”* or *“B cell,”* or *“pre-B cell.*” Remaining genes were then refined with reference sets in the Human Protein Atlas based on flow cytometry of immune subsets and single-cell RNAseq transcriptomics, https://www.proteinatlas.org/humanproteome/immune+cell (20). Genes that had B cell RNA blood cell lineage plus B cell type specificity on Human Protein Atlas were added to the “B-cell-related” genes list. Using the same approaches, (2) “Some-B-cell-relation” genes had some, but not exclusive, B cell lineage and/or cell type specificity. (3) “Non-B-cell-related” genes had neither B cell lineage or cell type specificity.

*T-cell* subset specificity was defined with a two-step process. First, R&D Systems Cell Markers by Category function created lists of Th1, Th2, Th17, and Treg markers^1^ (Bio-Techne). Then to address overlapping surface markers, th-express.org/ expression atlas (21) assigned a numeric score to a gene’s expression in Th17, Th1, Th2, and Treg (CD4+ spleen-resident Treg and induced Treg [iTreg]). B naïve and memory, and CD8 CTL and Treg subsets, are not defined in these datasets because they have few unique mRNAs. For instance, RNA coding for CD8 CTL markers such as granzyme and perforin is more highly expressed in NK cells (scRNAseq) and γδ T cells (flow cytometry plus RNA analysis); RNA for IFN-γ is shared with Th1 cells. The final gene lists were composed of T subset-specific genes with expression *z*-scores greater than expression in other T-cell subsets (*p* < 0.05).

A *monocyte* gene list was created using targets that were in R&D Systems’ monocyte surface marker database plus cell type specificity in healthy controls, according to the Human Protein Atlas.

### Statistical analysis

**Figure 2:**
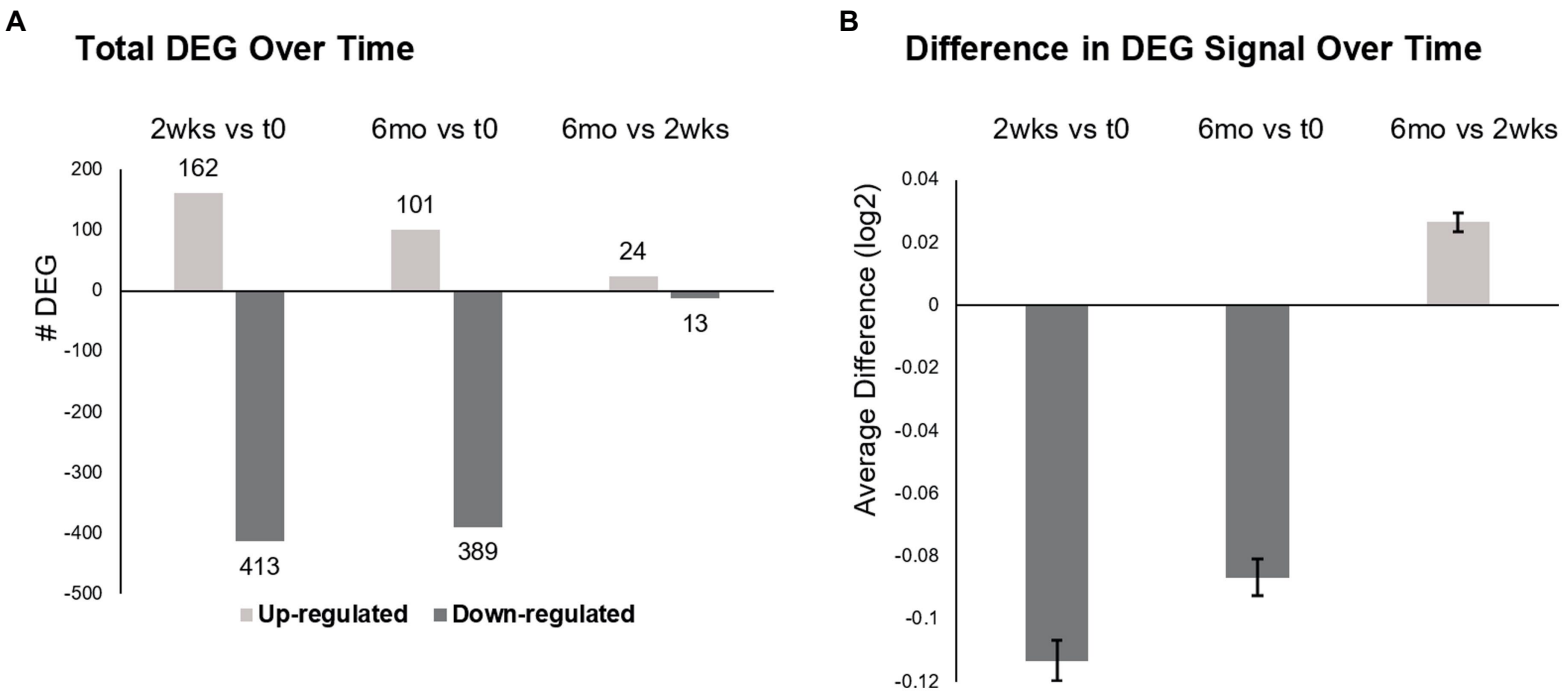
Differentially expressed genes (DEG) in MS PBMC at time 0, 2  weeks, and 6 months after ocrelizumab infusion. **(A)** Total number of DEG at 2  weeks post-ocrelizumab infusion vs. time 0 after 2-month prior therapy washout (575 DEG), at 6 months vs. time 0 (490 DEG), and at 6 months vs. 2  weeks (37 DEG). **(B)** The average difference in gene expression increases at 6  months vs. 2  weeks. Significance between these large numbers of DEG is high; for 0.5 vs. 0, *p* = 7 × 10^−67^; 6 vs. 0, *p* = 7 × 10^−49^; 0.5 vs. 6, *p* = 9 × 10^−18^, two-tailed paired *t*-test. **(A,B)** Based on 9,406 genes with Time *F*-Test <0.05, log2 scale. See text for calculations. Bars show mean ± SEM.

Differential gene expression was determined with the TAC Limma Bioconductor package (22). Pairwise ANOVA with repeated measures tracked longitudinal changes in gene expression for individual ocrelizumab-treated patients at time 0, 2 weeks, and 6 months. Change in gene expression was deemed significant if it passed both value of *p* < 0.05 and fold-change <−2 or > 2, more stringent than the usual |1.5| fold-change criterion. IPA pathways were scored by *z*-score and value of *p*. The number of upregulated vs. downregulated genes and paired protein changes were compared with 2 × 3 *X*^2^ matrix, vs. null hypothesis of no change in all targets. Subset-specific changed genes and intersubset expression (e.g., Th1 vs. Th2) were compared with a 2 × 2 *X*^2^ matrix (Figure 2).

Raw microarray data files are available in the NCBI Gene Expression Omnibus (GEO) repository, Accession #GSE pending acceptance.

## Results

### Total gene signatures in PBMC differ between pre- and post-ocrelizumab MS, clinically stable therapy-naïve and IFN-*β*-treated MS, and HC

At time 0, 2 months after washout of prior therapies, expression of 1,104 genes decreased, and 128 increased, compared to expression in HC (Figure 1A and Supplementary Table S1, gene list). Gene expression at time 0 was slightly decreased compared to stable therapy-naïve and IFN-*β*-treated MS (Figures 1B,C). There was further decrease 6 months after ocrelizumab treatment in all groups (Figures 1D–F). Reduced gene expression at time 0 in MS is likely due to lingering effects of prior MS therapy. Further analysis of the IFN-β control group is not repeated here. We have shown that IFN-*β*-1b normalizes gene expression, inducing transition from 8,800 dysregulated genes in untreated MS to only 300 during IFN-β therapy (19).

### Total gene expression in PBMC declines at 2  weeks after ocrelizumab treatment, but rises toward baseline by 6  months

Total DEG were largely downregulated at 2 weeks (72% decreased) and 6 months (79%) after the first infusion of ocrelizumab (Figure 2A). Six months after the initial infusions, there were 490 DEG compared to time 0, less pronounced than at 2 weeks (575 DEG). At 6 months compared to 2 weeks, 24 of 37 DEG were upregulated.

The degree of expression paralleled the number of DEGs. Average gene expression for each of 9,406 DEG was compared to change in expression over time (Time *F*-Test <0.05), by calculating averaged expression at 2 weeks minus expression at time 0 (Figure 2B). This process was repeated for 6 months vs. time 0, and for 6 months vs. 2 weeks. Average gene expression at 2 weeks diminished compared to time 0 (−0.11329, log2 scale) and remained negative at 6 months vs. time 0 (−0.08676). However, there was partial upregulation at 6 months vs. 2 weeks (+0.026535). Thus, there is widespread decrease in gene number and degree of expression after the initial half dose ocrelizumab, and these begin to return toward baseline by 6 months after an infusion.

### Gene expression differs between specific B-cell-related and non-B-cell-related genes

**Figure 3:**
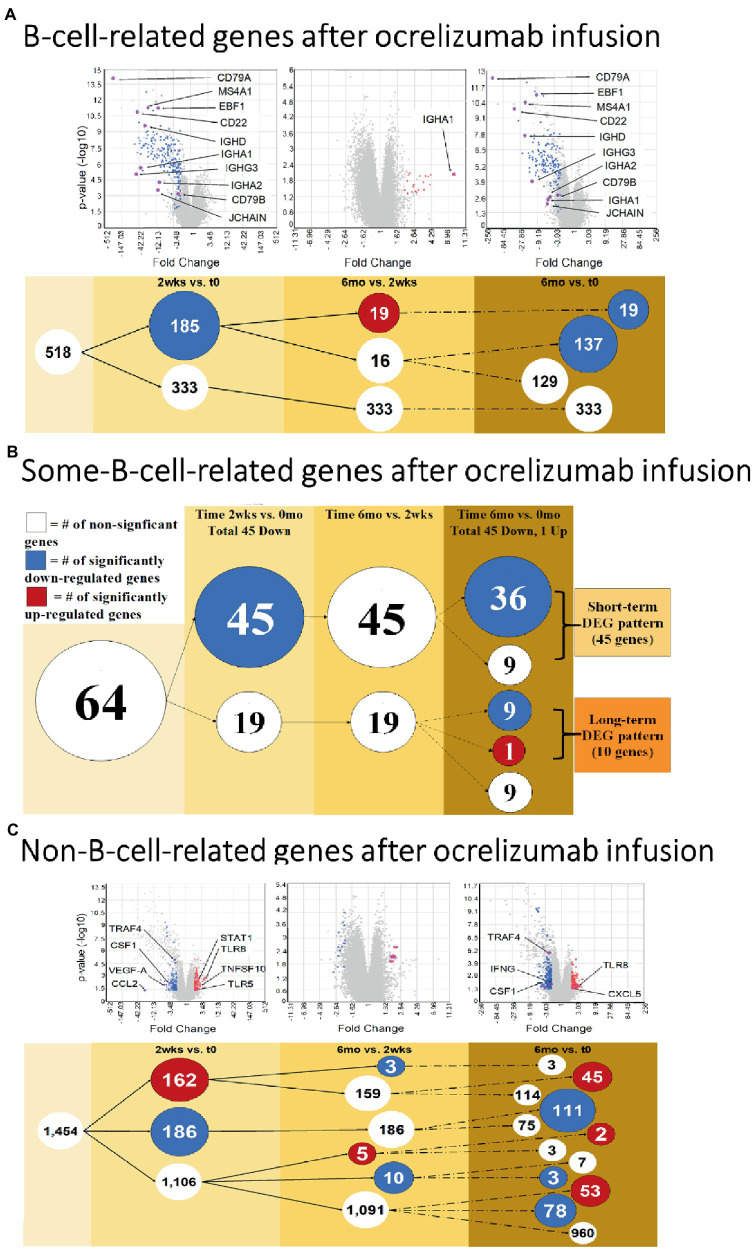
B cell and Non-B-cell gene expression in ocrelizumab-treated MS PBMC. **(A)** B-cell-related DEG decrease at 2  weeks and 6 months after infusion. Downregulation of gene expression predominates. **(B)** Some-B-cell-related gene expression in MS PBMC. Changes parallel **(A)** because loss of gene expression by B cells is the most visible effect of anti-CD20 B cell depletion. **(C)** Non-B-cell-related gene expression in MS PBMC. There is an increase of expression in some genes, unlike the B cell profiles. *Red*: genes pass FC > 2 and *p* < 0.05 criteria. Blue: genes pass FC < −2 and *p* < 0.05. White: genes do not pass FC > |2| and/or *p* < 0.05. Venn sphere and font size reflects number of DEG. Labeled genes are discussed in text; All genes are listed in Supplementary Tables S2–S4.

Since ocrelizumab profoundly depletes CD20+ B cells, we extracted signatures of B-cell-specific from non-B-cell-related genes within the PBMC population. At 2 weeks compared to time 0, 185 “B-cell-related genes” showed downregulated expression; none was upregulated (Figure 3A and Supplementary Table S2, gene list; See Methods for definitions). At 6 months compared to 2 weeks, expression of 19 of these genes was upregulated; all 19 were immunoglobulin-related.

Genes shared between B cells and non-B cells will also reflect B cell loss after extensive peripheral B cell depletion. Expression of genes in the “some-B-cell-relation” category was downregulated in 45 genes at 2 weeks vs. time 0. At 6 months vs. time 0, expression decreased in 45 genes (36 of which were also downregulated at 2 weeks vs. time 0), and one increased (Figure 3B and Supplementary Table S3, gene list).

“Non-B-cell-related” gene expression had a more variable trajectory after therapy, in contrast to the universal decrease in B cell gene expression. At 2 weeks vs. time 0, expression of 186 genes decreased, but 162 genes increased (Figure 3C and Supplementary Table S4, gene list). At 6 months vs. time 0, expression of 192 genes decreased and 100 increased. Expression in 53 of these genes was not increased at 2 weeks (55% of 1,106 DEG showed late induction. At 6 months vs. 2 weeks, expression of 13 genes decreased and 5 increased). Thus, changes in non-B-cell-related genes appear soon after infusion and are still present at 6 months.

### Expression of B-cell-related genes relevant to MS is downregulated after ocrelizumab infusion

**Figure 4:**
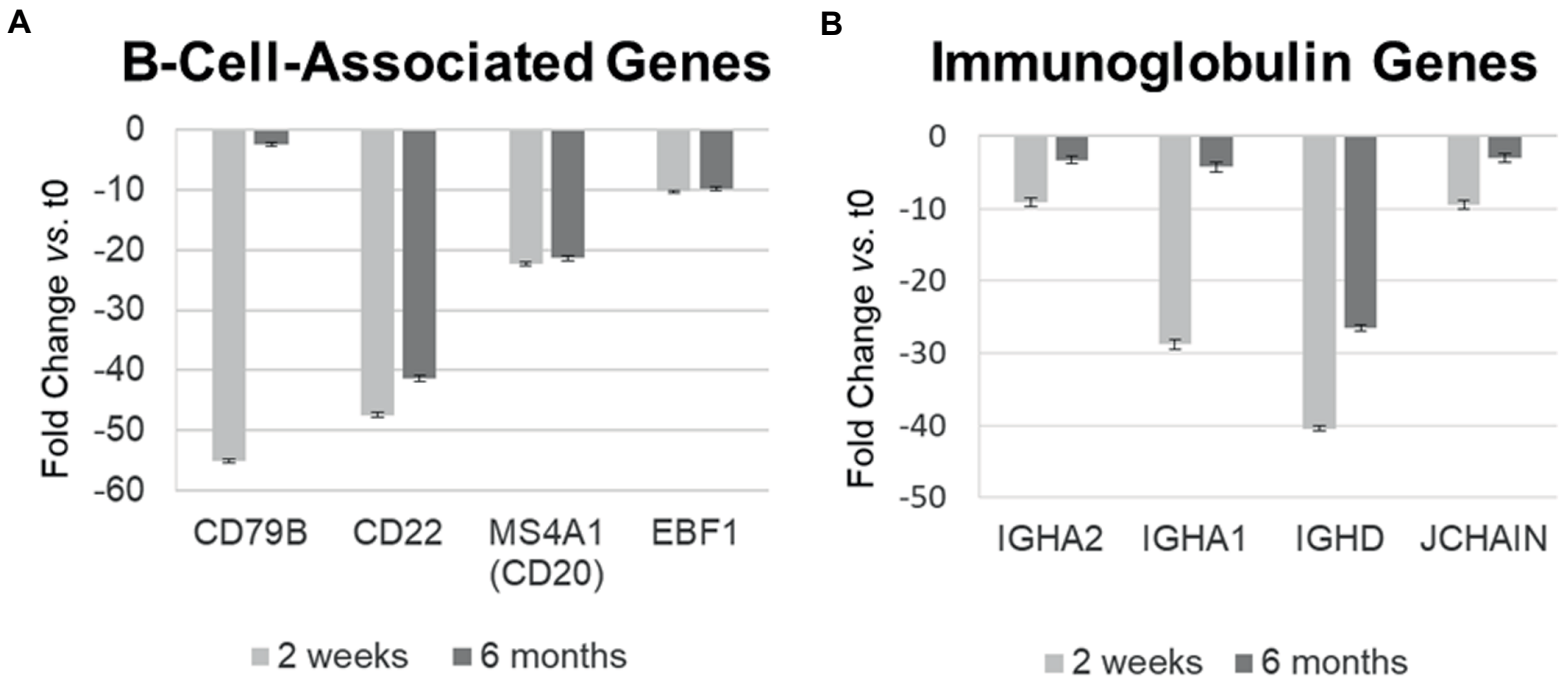
Differential gene expression over time in individual B-cell-related genes in MS PBMC at 2  weeks and 6 months after ocrelizumab infusion compared time 0. **(A)** B-cell-associated genes typically remain decreased at 2  weeks and 6 months after anti-CD20 infusion. All values are significantly below 0; CD22 and CD20 show biologically slight, but statistically significant recovery between 2  weeks and 6  months after infusion; *p* < 0.05 or less, two-tailed paired *t*-test. **(B)** Immunoglobulin-associated gene expression returns toward baseline at 6 months. All values are significantly below 0 and show statistically significant recovery between 2  weeks and 6  months. Fold-change at 2  weeks (light gray) and 6  months (dark gray). IGHA, IgA heavy chain; JCHAIN, Joining chain of multimeric IgA and IgM; other genes described in text. Bars show mean ± SEM.

Ocrelizumab downregulated multiple B-cell-expressed genes at 2 weeks, and many showed minimal return toward baseline by 6 months. Genes included *CD79A* (fold-change at 2 weeks vs. time 0 = −272; at 6 months vs. time 0 = −172; not shown in figure because of extreme scale of change) and *CD79B* (−55.0; and − 2.46) (Figure 4A). The CD79A/B heterodimer transmits an activating signal when antigen binds to the B cell receptor (BCR) in CD20+ B cells and in CD20-negative plasmablasts and plasma cells (23). Different RNA expression in genes coding for the two components of this heterodimer is unexplained, but CD79B is rate-limiting in heterodimer formation (24). *CD22* (mature B cells; inhibits signaling) rose from its nadir by 15% at 6 months. Importantly, downregulation persisted in other B cell genes, including *MS4A1* (CD20, late pro-B cells through memory cells; enhances BCR response), *CD19* (member of costimulatory complex of CD21 complement receptor, CD81, and CD225), *EBF1* (early B cell factor 1, regulates B cell commitment) (Figure 4A), and *CD80* (costimulatory molecule increased on B cells from therapy-naïve MS patients (9); fold-change = −1.40 at 2 weeks, and − 1.35 at 6 months).

Immunoglobulin-associated gene expression also decreased at 2 weeks, but in contrast, showed upregulation from its nadir by 6 months (Figure 4B). Genes included IgG, D, and A heavy chains, and the J chain that joins IgA and IgM subunits. Expression of the majority of B cell lineage and surface markers did not return toward baseline (Figure 4A). This suggests that residual CD20-negative and CD19-negative B cells (i.e., plasma cells) were stimulated to produce more Ig RNA.

### Some pro-inflammatory pathways are upregulated by ocrelizumab, despite downregulation of gene expression for B cell differentiation and development

Changes in gene expression reflect maturation and activation of immune subsets in PBMC, and can be inferred to reflect organ-specific changes. Although CNS expression was not studied, there is high correlation between tissues (25).

**Figure 5:**
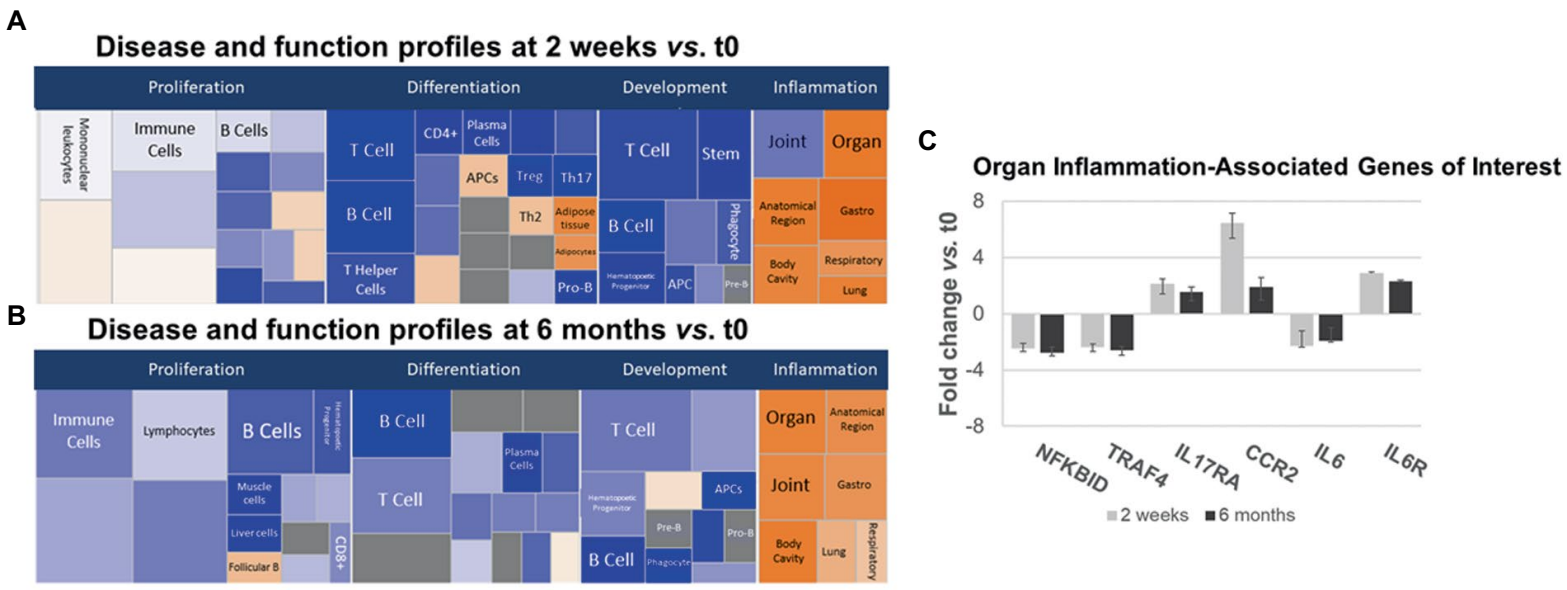
Ingenuity pathways analysis (IPA) disease and function pathways classification of immune-relevant effects of ocrelizumab in MS PBMC. Disease and Function analysis of gene expression in MS PBMC at 2  weeks **(A)** and 6  months **(B)** after infusion vs. t0. There is downregulation of B- and T-cell proliferation, differentiation, and development pathways, but upregulated inflammatory pathways. Klimt plot squares are sized by significance (−log10[value of *p* of fold-change]) and colored by *z*-score of the expression magnitude of predicted inhibition (blue) or activation (orange). **(C)** Organ-specific or region-specific inflammatory gene expression in PBMC from MS patients at 2  weeks and 6  months after ocrelizumab infusion compared to time 0. All are fold-change > |1.5|. Genes triggering the pro-inflammatory “organ-specific” result in **(A,B)** include upregulated pro-inflammatory *IL6R*, *IL17RA*, and *CCR2*, and downregulated anti-inflammatory *NFKBID* and *TRAF4*.

Gene expression in IPA Disease and Function pathways affecting B- and T-cell proliferation, differentiation, and development of immune cell subsets decreased 2 weeks after anti-CD20 infusion (Figures 5A,B, left and center, blue boxes). The decrease in proliferation profiles was pronounced in B cell pathways, but also appeared in Th1, Th17, and Treg pathways. In contrast, expression increased in differentiation pathways for adipocyte, Th2 cells, and antigen presenting cells at 2 weeks and for follicular B cells at 6 months. These pathways do not necessarily reflect inflammation or cytokine production.

Inflammatory organ-specific and region-specific gene pathways were overexpressed after ocrelizumab treatment (Figures 5A,B, right, orange), despite downregulated immune cell development pathways. Categorization is based on the peripheral immune signature of MS and shows commonality with other inflammatory and organ-specific diseases. CNS inflammation is not characterized but is likely to be reflected in peripheral immunity. Because anti-CD20 therapy depletes B cells, and no B cell genes increase at 2 weeks, this organ-specific inflammation arises from non-B-cell pathways. Genes with most-changed expression in pro-inflammatory pathways included upregulated *IL6R*, *IL17A* (Th17 cells), and *CCR2* (monocytes and Th1 cells), and downregulated anti-inflammatory *NFKBID* (NFKB inhibitor δ; present in many cells) and *TRAF4* (monocytes) (Figure 5C). Expression of *IL6* (naive and memory B cells) is downregulated 2-fold, whereas the IL-6 receptor gene (B cells and other PBMC), is upregulated 2–3 fold. Increased *IL-6R* expression may reflect compensation for low IL-6 levels, akin to denervation supersensitivity. We next explored T and myeloid cell gene expression in more detail.

### Ocrelizumab treatment increases Th1-linked gene expression, but has mixed effects on Th2, Th17, and Treg cell gene expression

**Figure 6:**
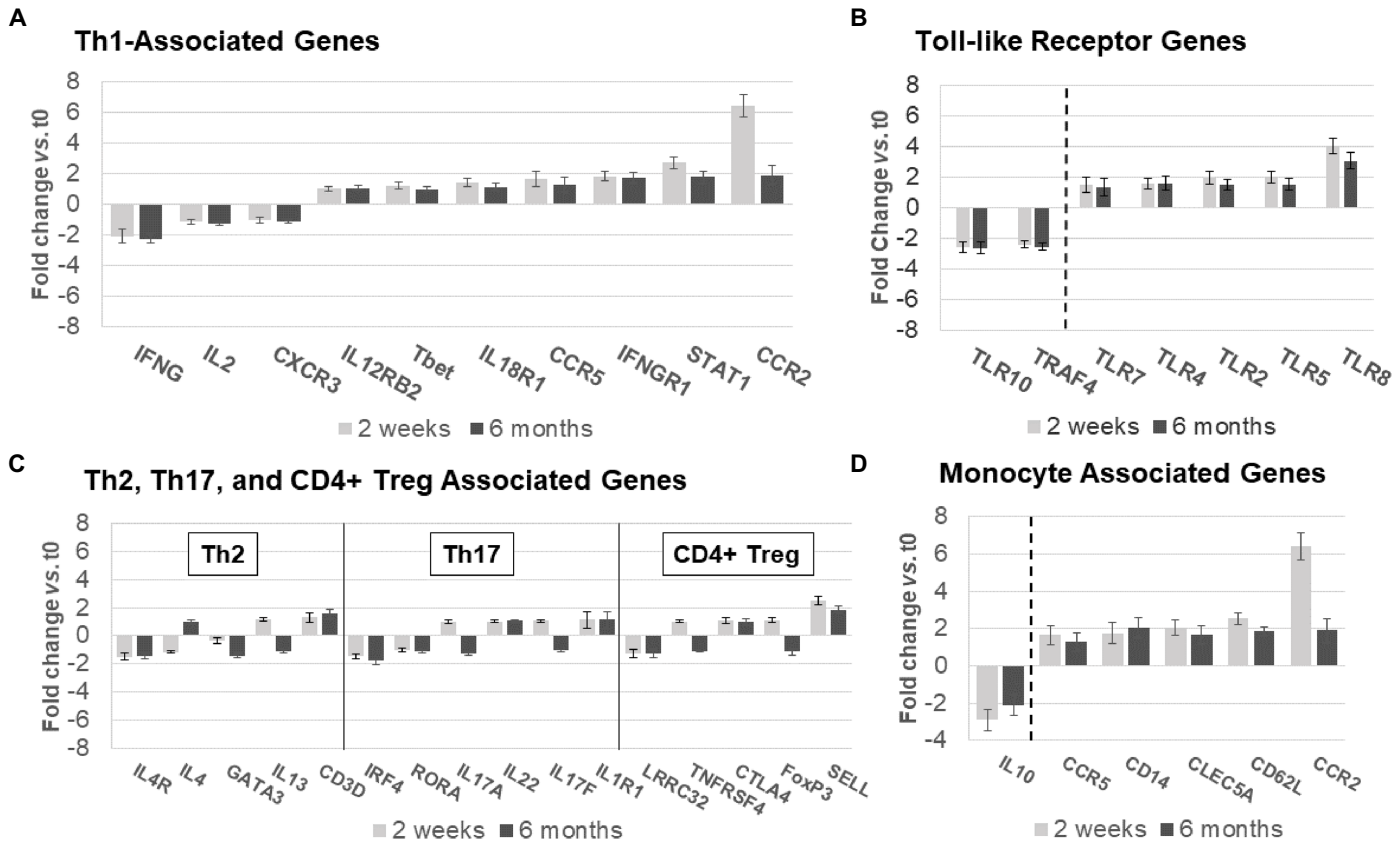
Expression of non-B-cell-related genes in MS PBMC at time 0, 2 weeks, and 6 months after ocrelizumab infusion. **(A)** Th1-associated gene expression is predominantly increased. IFN-γ, CCR5, IFNγR1, STAT1, and CCR2 have fold-change > |1.5|. **(B)** Th2, Th17, and CD4+ Treg-associated genes show minimal change. Only 3 have fold-change > |1.5| and none> |2|. **(C)** Toll-like receptor associated genes show strong upregulation in expression of pro-inflammatory genes, and downregulation of anti-inflammatory *TLR10* and *TRAF4*. All have fold-change > |1.5|. **(D)** Monocyte-associated genes also show strong upregulation, paired with decrease in expression of anti-inflammatory *IL10*. All have fold-change > |1.5|. Genes shown are those with greatest change in expression for each immune subset. Gene expression 2  weeks (light gray) and 6 months (dark gray) after ocrelizumab infusion vs. time 0. Deconvolution analysis of subsets is described in Methods. Bars show mean ± SEM.

Expression of genes coding for signaling proteins and effector molecules reflects the activation state of T cells. Th1 pathway expression predominated at 2 weeks (Figure 6A), with more *CCR5, STAT1*, *CD119* [IFN-γR1, linked to inflammation in MS (26)], and *CCR2* (in pro-inflammatory Th1 and myeloid cells), with only partial return to baseline by 6 months after ocrelizumab infusion (Figure 6A). A smaller group of Th1 genes had decreased expression 2 weeks and 6 months after infusion, including *IL-2*, *CXCR3* (on Th1 cells), and *IFNG* [coding for IFN-γ, a pro-inflammatory Th1 cytokine that causes MS exacerbations (27)]. Th1-specific transcription factors, *STAT4* and *TBX21* (*aka Tbet*), and lineage markers reflecting the number of T cells, did not change, paralleling Figures 5A,B.

Th2, Th17, and CD4+ Treg linked gene expression had no consistent change in direction at 2 weeks and 6 months after ocrelizumab therapy (Figure 6B). Stable expression of lineage transcription factors *Tbet/TBX21* (Th1), *RORα* (Th17), and *GATA3* (Th2) also suggests that numbers of these T-cell subsets are stable during ocrelizumab treatment.

### Ocrelizumab treatment upregulates expression of genes coding for TLR and innate immunity

Ocrelizumab treatment induced inflammatory toll-like receptor (TLR) signaling pathways (Figure 6C). *TLR* genes are expressed in innate immune cells (monocytes, macrophages, dendritic cells, and NK cells) that respond to pathogens and danger signals, and in turn control T and B cell adaptive immunity (28–31). Increased expression of pro-inflammatory *TLR2, TLR4, TLR5,* and *TLR8* was coupled with downregulation of anti-inflammatory *TRAF4* and *TLR10* genes (32, 33), likely facilitating a pro-inflammatory state in innate immune cells.

Monocytes and other immune cells regulate expression of some Th1 genes. Expression increased in pro-inflammatory monocyte genes, *CD14*, *CCR2*, *CCR5*, *CD62L*, and *APRIL*, coupled with decreased anti-inflammatory *IL-10* (Figure 6D). The latter is relevant in MS immune regulation because IL-10 inhibits production of inflammatory IFN-γ and TNF-α (34). Monocytes, which infiltrate the brain in MS, produce 15-fold more IL-10 protein than T cells on a per-cell basis (35). Overall, ocrelizumab appears to bias Th1 cells and innate immune cells toward a pro-inflammatory state.

### Serum immune and neuroprotective protein profiles change at 2  weeks and 6  months after ocrelizumab treatment

**Figure 7:**
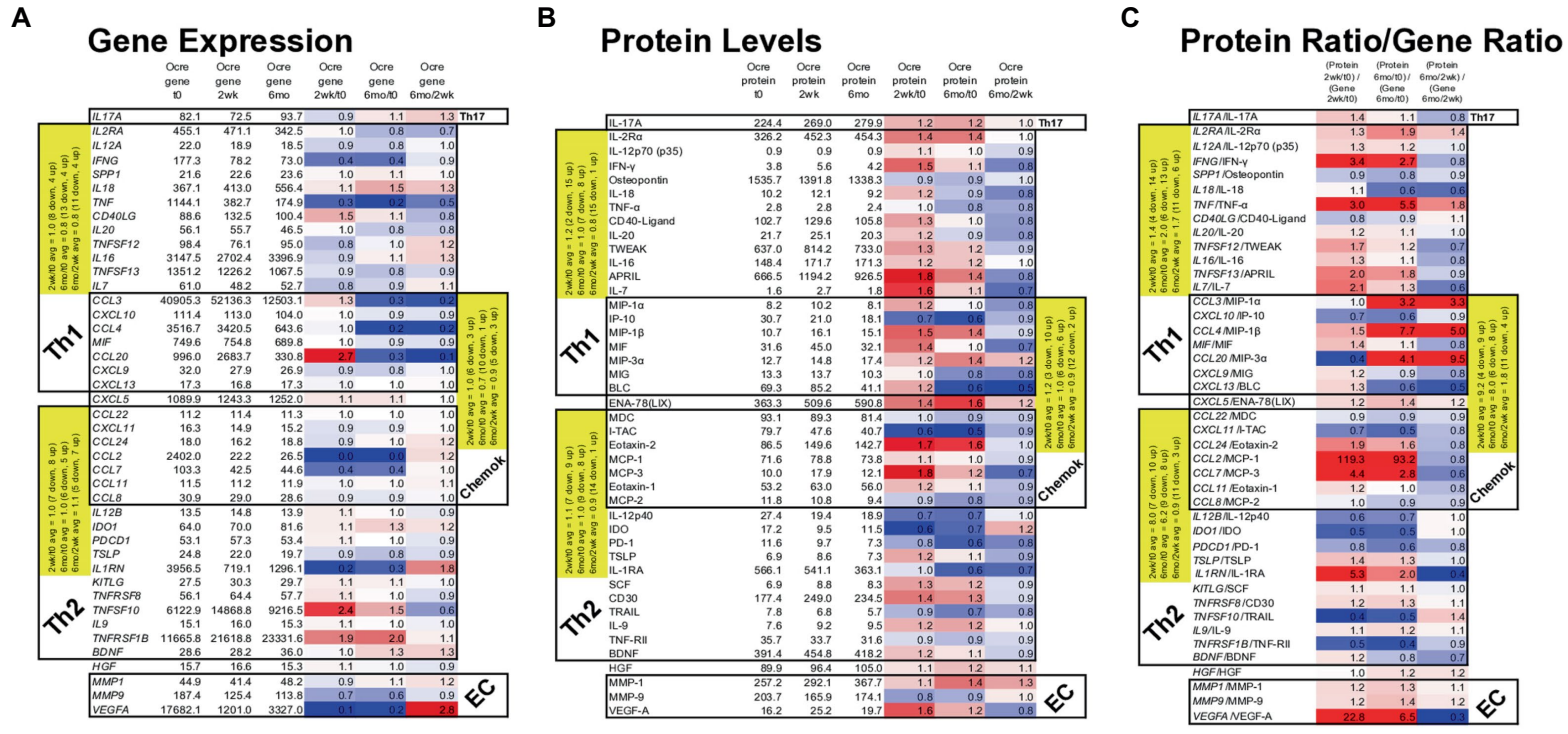
Gene expression in MS PBMC and paired serum protein levels of 43 MS-relevant targets at time 0, 2  weeks, and 6  months after ocrelizumab infusion. **(A)** Gene expression (raw signal, white) change = ratio of at 2  weeks/time 0, 6  months/time 0, and 6  months/2  weeks. **(B)** Serum protein level (pg/ml) change = level at 2  weeks/time 0; 6  months/time 0, and 6  months/2  weeks; repeated as in **(A)**. **(C)** Protein/gene ratio of changes = (protein level at 2  weeks/time 0)/(gene expression at 2  weeks/time 0); repeated as in **(A)**. Red = protein level change > gene expression change (i.e., ratio > 1.0); blue = protein change < gene change (ratio < 1.0); white = equivalent protein and gene change (ratio = 1.0). Average changes of grouped Th1, Th2, chemokine, and extracellular matrix (EC) markers over time are in yellow. Genes in italics; proteins in upright font. Red = increase; blue = decrease. The Th1 cluster includes myeloid and other cell products that induce Th1 cells and which are inflammatory in the context of MS. Similarly, Th2 includes proteins that are anti-inflammatory in the MS context.

After ocrelizumab treatment, expression of genes in PBMC was compared to 43 paired MS-relevant serum immune and neurotrophic proteins (Figure 7A). The Th1 cluster in these curated proteins includes myeloid cell products that induce Th1 cells, inflammatory in the context of MS (16, 17). The Th2 and BDNF group are anti-inflammatory in the MS context (36).

Nineteen Th1, Th17, and chemokine genes coding for pro-inflammatory proteins in MS (“Th1” in Table) decreased in expression at 2 weeks and 6 months after ocrelizumab infusion (Figure 7A). For 18 Th2 and chemokine genes, anti-inflammatory in the MS context (“Th2” in Table), there was no change in in direction of expression. After excluding Th2-linked chemokines, 11 Th2 genes rose at 2 weeks and 4 rose at 6 months. Th1 genes had a relative rise vs. Th2 genes at 2 weeks. The selected genes in Figure 7A change in the same direction as the larger group of non-curated total genes in Figure 6, where expression of Th1 and chemokine genes tended to diminish, but Th2 genes to increase after ocrelizumab exposure.

Proteins contrasted to gene expression. 19 pro-inflammatory T cell and monocyte proteins increased at 2 weeks and 9 rose at 6 months after anti-CD20 infusion (Figure 7B). Eighteen Th2-linked proteins showed minimal overall change at 2 weeks and 6 months. Th1 protein levels rose compared to Th2 proteins at 2 weeks. As a measure of translational impact, the ratio of the change in protein over the change in gene expression (Figure 7C), usually paralleled the changes in protein levels after anti-CD20 infusion. Thus, pro-inflammatory proteins increase after ocrelizumab infusion.

Individual protein/gene pairs suggested a pro-inflammatory effect of ocrelizumab. *CD40LG* (CD40 ligand) and its protein are upregulated at 2 weeks and return to baseline by 6 months (Figure 7). CD40-ligand is pro-inflammatory and expressed on T cells. Its receptor, CD40, is expressed on B cells and professional antigen presenting cells (APC). Increased expression of CD40-ligand on T cells could enhance T cell/APC interaction and inflammation.

VEGF-A is a pro-inflammatory growth factor that enhances vascular endothelial cell migration and proliferation; it also attracts Tregs. *VEGFA* gene expression decreased at 2 weeks, but increased at 6 months (Figure 7A). In contrast, the effector protein, VEGF-A, increased at 2 weeks and decreased at 6 months (Figure 7B), suggesting a less inflammatory serum environment. Discrepancy between PBMC gene expression and serum protein levels may be from non-lymphoid sources of VEGF, such as reactive astrocytes and renal glomerular podocytes.

Monocytes produce APRIL (*TNFSF13*), BAFF/BLyS (*TNFSF13B*), IL-12, and IDO. APRIL, a pro-inflammatory B cell growth factor, increases 2 weeks and remains elevated 6 months after B cell depletion. APRIL binds to three receptors, BAFF-R (on peripheral B cells, not plasma cells), TACI (memory B cells, plasma cells), and BCMA (plasma cells). CD20-plasma cells are not depleted by anti-CD20 therapy and will respond to APRIL. *TNFSF13* (APRIL) levels correlate negatively with its receptors’ RNA levels. Two weeks after B cell depletion, expression decreased for *BAFFR* and *TACI*, and trended down for *BCMA* (Figure 7A). BAFF protein has 10-fold slower clearance than TNF-α and TRAIL, which are detectable in our assays. BAFF protein, however, was often not detectable in serum and the *BAFF/TNFRSF13C* gene did not change. In the patients in whom BAFF was detectable, average serum BAFF decreased at 6 months, suggesting absorption by target cells. BAFF binds predominantly to BAFF-R on circulating CD20+ B cells (depleted by anti-CD20 therapy), and on bone marrow IgM+ B cells, splenic transitional, marginal zone, and follicular B cells, and lymph node memory B cells (37). BAFF may be undetectable because of absorption by repleting CD20+ cells and remaining CD20-negative B cells and thymic Treg (38).

IL-12 p35 (*IL12A* gene) and IL-12 p40 (*IL12B* gene) form the IL-12p70 heterodimer, which induces pro-inflammatory Th1 cells. *IL12A* decreases after anti-CD20 infusion, but *IL12B* increases and then returns to baseline by 6 months (Figure 7A). IL-12p70 protein levels change minimally (Figure 7B). The IL-12p40 homodimer is anti-inflammatory and counteracts pro-inflammatory IL-12p70 (39). IL-12 p40, however, decreases after infusion, leading to a net pro-inflammatory IL-12 state.

*IDO* gene and protein expression decreases 2 weeks and 6 months after ocrelizumab infusion (Figure 7). IDO encourages regulatory T-cell differentiation. The decrease in immunoregulatory IDO differs from the many pro-inflammatory effects above.

## Discussion

Ocrelizumab therapy depletes CD20+ mature and memory B cells, but preserves bone marrow B cell precursors and antibody-secreting plasma cells. We find that anti-CD20 therapy decreases B cell gene expression at 2 weeks, but expression partially recovers at 6 months, especially for immunoglobulin-related RNA. Beyond the effects on B cells, ocrelizumab increased markers of Th1 cell inflammation, and increased gene expression in innate myeloid cells, including antiviral toll-like receptor (TLR) genes. This suggests that T cells and monocytes can partially compensate for deleted B cells. These findings broaden understanding of the mechanism of action of anti-CD20 therapy in MS, and have implications for timing of vaccinations and for the safety profile of B cell depleting therapies.

B cell gene expression decreases 2 weeks after anti-CD20 infusion and returns toward baseliner by 6 months. These B cell genes code for immunoglobulins and pro-inflammatory proteins, such as TNF-α and GM-CSF, produced by activated plasmablasts (40, 41). This upregulation suggests activation of circulating CD20-negative B cells, such as plasmablasts and plasma cells. The appearance of small numbers of newly differentiated CD20+ B cells is unlikely. CD19+ B cells, 6 months after ocrelizumab infusion in controlled clinical trials, remain at 0–1 cells/μL blood, compared to 230 B cells/μL at baseline (1). Even if there are no measurable circulating B cells, there is repopulation of autoreactive B cells in lymph nodes in the experimental autoimmune encephalitis model of MS (42). Local B cell activation in bone marrow, secondary lymphoid organs, and perhaps the meninges, would be missed in analysis of circulating PBMC (3). Moreover, repopulating B cells are less mature and more activated in MS (43). As a second possible therapeutic correlate, reduced expression of RNA coding for IgA1, IgA2, and J chain (Figure 4B) may reflect depletion of potentially pathogenic IgA+ B cells which mediate inflammatory diseases including MS, and traffic to the CNS (1, 7, 44). No patients herein had pulmonary or mucosal infections, but IgA serum levels in a larger series would be of interest.

The rise in immunoglobulin genes at 6 months has clinical implications. Anti-CD20 depletion of antigen-specific B cells will prevent critical interactions with T follicular helper cells in lymph nodes and diminish vaccine responses (45). This may be amplified during COVID-19 infection due to disruption of lymph node architecture (46, 47). Anti-CD20-treated patients, vaccinated 3 to 4.5 months after infusion, generate anti-influenza antibodies at 82% of levels seen in untreated or IFN-β-treated MS (48). Nonetheless, only 50% of vaccinated patients have neutralizing antibodies against the COVID-19 spike protein after a second ocrelizumab dose (49). The ideal time for vaccinations may be at 3 weeks before the next infusion of anti-CD20 to take advantage of falling drug concentrations and increasing antibody responses at 6 months, and to avoid the effects of co-administered glucocorticoids and a transient cytokine storm from B cell death at anti-CD20 infusion (50). Additionally, T-cell immunity is robust and prolonged during anti-CD20 therapy (discussed below). Resurgence of immunoglobulin and other B cell-related genes by 6 months also suggests that reinfusion of ocrelizumab for control of MS is warranted approximately half a year after the first injections.

T-cell differentiation genes decrease for 6 months after ocrelizumab infusion (Figures 5A,B). The mechanism is most likely to be a consequence of B cell depletion. There is a transient 5% fall in peripheral blood CD3+ and CD8+ T cells following ocrelizumab therapy (1, 51) from immune cell redistribution due to concomitant glucocorticoid infusion (52), or from elimination of a small number of pro-inflammatory CD8 T cells with low levels of CD20 on their surfaces (13–15). T-cell numbers return to normal levels within weeks (1, 51), yet gene expression reflecting T-cell differentiation and proliferation remained low at 6 months. This suggests there is less drive for T-cell differentiation in the absence of CD20+ B cells, but there is activation of residual T cells.

At 2 weeks and 6 months after anti-CD20 infusion, gene expression increases in pro-inflammatory Th1 and myeloid cells. Patients herein were studied before the COVID-19 pandemic. But as a corollary for a compensatory role of enhanced T-cell function during B cell therapy, anti-COVID vaccinations in MS induce (1) flu-like symptoms from release of type I IFNs, lymphotoxin, and TNF-α and cytokines from myeloid cells (41, 53), (2) a rise in CD8+ cytolytic T cells, (3) clear CD8 and CD4 T-cell responses to COVID antigens (49, 54), (4) a fourfold increase in IFN-γ-secreting T cells (55), and (5) effective T-cell responses after a third booster vaccination (56). This indicates that serum immunoglobulin titers reflect only part of the immune repertoire, as T cell and innate immunity is preserved and responds to COVID vaccinations.

The immune power vacuum from B cell depletion appears to induce compensatory changes in other arms of immunity such as pro-inflammatory chemokine, type I interferon, IL-6, TLR, and classical monocyte signaling pathways. A homeostatic shift toward innate immunity and maintenance of T-cell immunity may explain why clinical trials and post-marketing data in ocrelizumab-treated patients show only slightly more frequent infections compared to their untreated counterparts, unexpectedly low rates of progressive multifocal leukoencephalopathy (PML), and no cancer signal (1, 57).

Serum protein levels as biomarkers may be more relevant than RNA expression. Here, changes in RNA expression were up to 100-fold in B cell genes, but less dramatic in T and innate cell gene expression. Relevant T and innate cell protein products did change with therapy. At 6 months after infusion, serum showed more Th1 proteins and pro-inflammatory monokines, and lower Th2-linked proteins (Figure 7B). Moreover, for some targets such as VEGF-A and IDO, changes in gene and protein levels in circulating, resting PBMC did not correlate (Figures 7A,B). This may be because RNA half-lives are usually shorter than protein half-lives (35), mixed immune subpopulations within PBMC, and because there are differences in post-transcriptional RNA and protein half-lives from 3′ regulation by lncRNA or poly-A tails (58). Finally, gene expression in circulating PBMC may not reflect the blood milieu, due to secretion of proteins from non-PBMC sources and protein absorption by target cells, and alternatively-spliced protein isoforms that are not detected by assay antibodies. RNA and protein correlations are seen best with highly-expressed chemokines (19). *In vitro* activated PBMC genes and protein supernatants show stronger but imperfect correlations (59). This is likely because immune cell subpopulations within PBMC have different post-transcriptional regulation of gene expression; e.g., IFN-β shortens the half-life of IL-10 mRNA in monocytes, but prolongs the half-life in T cells (35). Importantly, slight changes in an immune subset ratio or in numbers of activated cells can have significant consequences (60). Many of the targets identified herein, including those with subtle but significant changes, are likely to have relevance for understanding disease mechanisms in MS and for clinical and therapeutic monitoring.

This work is limited because (1) Data collection was not monthly and only extends to 6 months. Longer-term data may detect additional changes in inflammatory or regulatory pathways. For instance, CD8 + CD28- Treg numbers rise from subnormal before anti-CD20 therapy to supranormal levels after more than 6 months of therapy (12). (2) The majority of the ocrelizumab group had recently changed therapy. Switching from other therapies reflects clinical practice, but may have affected T and B cell baseline function compared to changes in therapy-naïve patients. (3) Patients were 50 years old compared to 37 in the RRMS and 45 in the PPMS pivotal trials of ocrelizumab (1, 2). Although we did not see age effects in gene expression, early inflammatory MS compared to late less-inflammatory MS could have different gene expression signatures and immune subset repopulation. (4) The sample size is small compared to cohorts in clinical trials and subtle effects of demographic differences and form of MS could have been missed. However, the paired longitudinal design allows internal replication and increases statistical power. Paired analysis of high quality RNA with thousands of targets/biomarkers (with statistical correction) contrasts with large clinical trials which have only 2–3 clinical outcome measures. (5) PBMC were used instead of whole blood. This was intentional, as PBMC contain the most MS-relevant leukocyte subsets, and whole blood adds 15-fold more RNA from neutrophils and reticulocytes, increasing bioinformatic noise (19). Sequencing after flow cytometry separation into subsets such as CD4 T cells (memory, naïve, Treg), CD8 T cells (CTL, Treg) (12), B cells, NK cells, and monocytes is possible, but requires massive resources. Single cell sequencing (scRNAseq) overcomes this limitation and is a good approach for studying B naïve and memory cells and CD8 subsets, but sequencing depth is much lower than with microarrays. We therefore used deconvolution analysis of the large number of genes to evaluate immune subsets. (6) This paper relies on stringent statistical benchmarks, such as a fold-change greater than |2|, instead of the usual |1.5|. While this strengthens our findings, it may prevent detection of subtle changes in expression of some genes. (7) Some individuals may have had return of circulating B cells at 6 months. We used the same infusion protocol as in the pivotal trials, where CD19 B cells were absent or barely detectable before the 6-month infusion in all ages, and in RRMS and PPMS (1, 2). In support, the current data show that CD19 and CD20 RNA levels remain at very low levels 6 months after infusion. (8) Serum protein levels are much lower than seen with *in vitro* activation, but do reflect the blood milieu in MS. (9) Finally, the proteins in Figure 7 were curated for MS relevance. However, immune regulation and inflammation in MS is likely to include proteins not detectable in multiplex assays (See genes in Figure 6 and Supplementary Tables S1–S4).

In summary, B cell depletion had wide effects on gene expression in immune cells. Gene expression in non-B cells differed from the profile in all PBMC and in B cells. We found that anti-CD20 therapy downregulates gene signatures in PBMC at 2 weeks and 6 months after starting infusions. B-cell-related genes account for much of this downregulation, which is likely linked to the 50% increase in nasopharyngitis, upper respiratory tract infections, and herpes infections compared to IFN-β, to potential long-term risk, and to doubling of COVID severity (1, 2, 61). However, expression of some B cell genes returns toward baseline by 6 months. This provides a rationale for redosing of anti-CD20 therapy at 6 months, and may explain “wearing-off” effects. Also, vaccine-induced antibody responses may be strongest around 5 to 6 months after infusions, when B cell gene expression begins to return. Despite B-cell downregulation, T cell and innate immune TLR signaling and classical monocyte pathway expression are maintained or upregulated in organ-specific inflammatory pathways and in PBMC. Upregulation of T-cell and innate pathways may enhance vaccine responses even when immunoglobulin titers are suboptimal.

## Data availability statement

The data presented in the study are deposited in the GEO repository, https://www.ncbi.nlm.nih.gov/geo/, accession number GSE228330.

## Ethics statement

The studies involving human participants were reviewed and approved by the University of Chicago Institutional Review Board. The patients/participants provided their written informed consent to participate in this study.

## Author contributions

XF and AR: study concept and design. CF, JS, QH-P, XF, and AR: data acquisition and analysis. CF, JS, QH-P, XF, and AR: drafting manuscript and figures. All authors provided substantial contributions to the conception and design of this work, drafting and revising the manuscript critically for important intellectual content, provided approval for publication of the content, and agreed to be accountable for all aspects of the work in ensuring that questions related to the accuracy or integrity of any part of the work are appropriately investigated and resolved.

## Funding

This research was funded by unrestricted grants from Roche-Genentech # ML4104 and Joe Griffin, and a stipend from the Biological Sciences Collegiate Division Research Endowments at the University of Chicago.

## Conflict of interest

XF has received unrestricted research support from Bayer, Biogen, BMS, Mallinckrodt, Merck-Serono, and Novartis, which produce drugs for the treatment of MS, some of which are anti-CD20 therapies, and Roche-Genentech, which produces rituximab and ocrelizumab studied in this paper. AR has received unrestricted research support from Bayer, Biogen, BMS, Roche-Genentech, Mallinckrodt, Merck-Serono, and Novartis, and is a consultant for Bayer, Biogen, Merck-Serono, Novartis, Roche-Genentech, and TG therapeutics.

The remaining authors declare that the research was conducted in the absence of any commercial or financial relationships that could be construed as a potential conflict of interest.

## Publisher’s note

All claims expressed in this article are solely those of the authors and do not necessarily represent those of their affiliated organizations, or those of the publisher, the editors and the reviewers. Any product that may be evaluated in this article, or claim that may be made by its manufacturer, is not guaranteed or endorsed by the publisher.

## Footnotes

^1^ rndsystems.com/resources/cell-markers/immune-cells

## Abbreviations

CC: Chemokine
DEG: Differentially-expressed gene
EC: Endothelial cell
IFN: Interferon
ISG: IFN-stimulated gene
MMP: Matrix metalloprotease
MS: Multiple sclerosis
NP: Neuroprotective/neurotrophic
PBMC: Peripheral blood mononuclear cells
PCA: Principal component analysis
TLR: Toll-like receptor
