# Corrigendum: Adaptive and innate immune responses in multiple sclerosis with anti-CD20 therapy: gene expression and protein profiles

**DOI:** 10.3389/fneur.2023.1245622

**Published:** 2023-08-08

**Authors:** Chloe C. Fong, Julian Spencer, Quentin Howlett-Prieto, Xuan Feng, Anthony T. Reder

**Affiliations:** Department of Neurology, University of Chicago Medicine, Chicago, IL, United States

**Keywords:** anti-CD20 therapy, B cell, multiple sclerosis, ocrelizumab, T cell, TLR

In the published article, there was an error. The level of CD79B RNA at 2 weeks was typed incorrectly in the text.

A correction has been made to **Results**, “*Expression of B-cell-related genes relevant to MS is downregulated after ocrelizumab infusion*,” paragraph 1. This sentence previously stated:

“Genes included *CD79A* (fold-change at 2 weeks vs. time 0 = −272; at 6 months vs. time 0 = −172; not shown in figure because of extreme scale of change) and *CD79B* (−2.47; and – 2.46) (Figure 4A).”

The corrected sentence appears below:

“Genes included *CD79A* (fold-change at 2 weeks vs. time 0 = −272; at 6 months vs. time 0 = −172; not shown in figure because of extreme scale of change) and *CD79B* (−55.0; and – 2.46) (Figure 4A).”

The authors apologize for this error and state that this does not change the scientific conclusions of the article in any way. The original article has been updated.

